# Self-delivery of TIGIT-blocking scFv enhances CAR-T immunotherapy in solid tumors

**DOI:** 10.3389/fimmu.2023.1175920

**Published:** 2023-06-09

**Authors:** Fan Yang, Fan Zhang, Feng Ji, Jiannan Chen, Jun Li, Zhengliang Chen, Zhigang Hu, Zhigang Guo

**Affiliations:** ^1^ Jiangsu Key Laboratory for Molecular and Medical Biotechnology, College of Life Sciences, Nanjing Normal University, Nanjing, China; ^2^ CAR-T R&D Department, Nanjing Blue Shield Biotechnology Co., Ltd., Nanjing, China

**Keywords:** MSLN, TIGIT, immunotherapy, CAR-T cell, solid tumors

## Abstract

Chimeric antigen receptor T cell therapy has become an important immunotherapeutic tool for overcoming cancers. However, the efficacy of CAR-T cell therapy in solid tumors is relatively poor due to the complexity of the tumor microenvironment and inhibitory immune checkpoints. TIGIT on the surface of T cells acts as an immune checkpoint by binding to CD155 on the tumor cells’ surface, thereby inhibiting tumor cell killing. Blocking TIGIT/CD155 interactions is a promising approach in cancer immunotherapy. In this study, we generated anti-MLSN CAR-T cells in combination with anti-α-TIGIT for solid tumors treatment. The anti-α-TIGIT effectively enhanced the efficacy of anti-MLSN CAR-T cells on the killing of target cells *in vitro*. In addition, we genetically engineered anti-MSLN CAR-T cells with the capacity to constitutively produce TIGIT-blocking single-chain variable fragments. Our study demonstrated that blocking TIGIT significantly promoted cytokine release to augment the tumor-killing effect of MT CAR-T cells. Moreover, the self-delivery of TIGIT-blocking scFvs enhanced the infiltration and activation of MT CAR-T cells in the tumor microenvironments to achieve better tumor regression *in vivo*. These results suggest that blocking TIGIT effectively enhances the anti-tumor effect of CAR-T cells and suggest a promising strategy of combining CAR-T with immune checkpoints blockade in the treatment of solid tumors.

## Introduction

Chimeric antigen receptor (CARs) T cells therapy is emerging as a hot spot for cancer immunotherapy. CAR-T cells are genetically engineered to express antigen-specific T cells that recognize and eliminate specific cancer cells independent of major histocompatibility complex (MHC) molecules (1, 2). CAR consists of intracellular signaling and transmembrane (TM) structural domains and extracellular single-chain variable fragments (scFvs) (3). These scFvs are hinge-linked light chain (VL) and heavy chain (VH) variable regions that specifically recognize and bind tumor-associated antigens (TAA) (4). As an emerging therapy after chemoradiotherapy, it has become an important treatment for attacking cancers due to its advantages such as MHC restriction and etc. CAR-T cell therapy has achieved remarkable therapeutic results on a variety of tumors, especially in hematologic tumors, but its therapeutic results in solid tumors are limited (5). The main limitations of CAR-T cells are the limited available target antigens, their vulnerability in the tumor microenvironment (TME), insufficient tumor killing capacity and low persistence (6). In the TME, tumors can evade immune-mediated recognition through multiple immune escape mechanisms thereby attenuating the killing ability of immune cells (7). Under chronic tumor antigen exposure, T cell dysfunction/dysregulation and upregulation of various checkpoint inhibitory receptors that limit T cell survival and function reduce tumor clearance (8–10). Interactions between immune cell types and non-tumor cells within the TME clearly affect tumor progression, invasion, and metastasis (11). Therefore, the question of how to attenuate the inhibitory effect of suppressive immune checkpoints in the TME has become a pressing need.

Mesothelin (MSLN) is normally expressed on the surface of mesothelial cells. It was found that MSLN is also overexpressed in a wide range of solid tumors (12). Due to its differential expression between cancer and normal tissues and its role in tumorigenesis, MSLN can be considered as a potential target for cancer immunotherapy (13–16). However, achieving a broader therapeutic application of CAR-T cells requires a multi-layered approach to improve efficacy and safety (6, 17). An increasing number of studies have shown that mesothelin plays an important role in the promotion of tumorigenesis and progression, although its function in physiological situations is not yet clear (18). Studies have shown that MSLN can promote tumor proliferation, metastasis, and resistance to chemotherapy. Since MSLN is a highly specific antigen in several cancers, CAR-T therapy has been considered to be a promising strategy for the treatment of these cancers.

T cell Ig and immune receptor tyrosine inhibitory motif (ITIM) structural domains (TIGIT) acts as an immune checkpoint that is highly expressed on the surface of natural killer cells (NK) and T cells, significantly limiting anti-tumor and other CD8^+^ T cell-dependent chronic immune responses (19–21). TIGIT belongs to the immunoglobulin superfamily, which consists of an extracellular immunoglobulin variable region (IgV) structural domain, a type I transmembrane structural domain and an intracellular structural domain with a classical ITIM and an immunoglobulin tyrosine tail (ITT) motif (22). CD155 is a high-affinity ligand of TIGIT (23). Once CD155, which is highly expressed on tumor surface, binds to TIGIT on NK and T cells surface, its killing effect on tumor cells is inhibited (24). The blocking of TIGIT/CD155 interaction is a promising approach in cancer immunotherapy (25). Many literatures describing the inhibitory immune test site TIGIT have shown that TIGIT-blocking antibody can be used in a variety of tumor treatments and are associated with T cell infiltration (26–28). However, it is still unknown whether the combination of TIGIT-related antibodies and CAR-T can achieve good efficacy since no study has been established.

For this study, we designed anti-MSLN CAR-T cells in combination with α-TIGIT antibody (anti-α-TIGIT) to treat the target cells. We found that anti-α-TIGIT effectively blocked TIGIT on the surface of CAR-T cells to a certain extent and increased the release of cytokines in anti-MSLN CAR-T cells to enhance the killing of target cells *in vitro*. Additionally, we genetically modified anti-MSLN CAR-T cells to have the ability to secrete anti-α-TIGIT scFvs for the long term. We found that the anti-α-TIGIT scFvs expression and secretion could interrupt the interaction of TIGIT with its ligand CD155, therefore enhanced CAR T cells infiltration and activation to promote tumor regression *in vivo*. In summary, our study demonstrated that blocking TIGIT effectively enhances the anti-tumor effect of CAR-T cells, thereby suggesting a promising strategy for the treatment of solid tumors by combining CAR-T cells with immune checkpoint blockages.

## Materials and methods

### Cell culture

Peripheral blood mononuclear cells (PBMCs, TPCS#PB025C) were purchased from the miles-bio of Shanghai. Normal human embryonic kidney cell line HEK293, T cell leukemia cell line NFAT-Jurkat, human cervical cancer cell lines Hela, human ovarian cancer cell lines (Skov3) were purchased from the American Type Culture Collection. T lymphocytes were maintained in T cell growth medium (TCGM): X-VIVO 15% Serum-free Hematopoietic Cell Medium (Lonza, Switzerland) supplemented with 5% FBS 2 ng mL^-1^ human recombinant IL-2 (100U mL^-1^, Sigma, Germany). HEK293 were maintained in Dulbecco’s modified Eagle’s medium (DMEM, Gibco, Grand Island, NY, USA). NFAT-Jurkat and Hela were maintained in RPMI 1640-media (Gibco, Grand Island, NY, USA). Skov3 were maintained in McCoy’s 5A medium (Gibco, Grand Island, NY, USA). GFP- and luciferase-expressing Hela (Hela-GL) and Skov3 cells (Skov3-GL) were generated by transfection of Hela and Skov-3 cells with lentiviral supernatant containing luciferase-2A-GFP. All cells were cultured in recommended medium supplemented with 10% FBS (Gibco) in a 10% CO_2_ incubator.

### CARs design and lentivirus packaging

The amino acid sequence of the human MSLN antibody was screened previously in the laboratory. The anti-MSLN scFv used originated from P4-scFv.The TIGIT-blocking scFv was originated from patent (Patent No.US20160176963A1). CAR constructs were synthesized and cloned into the pCDH lentiviral plasmid backbone with a human CMV promoter. A lentiviral vector containing a CAR consisting of the anti-MSLN scFv, CD8 hinge region, CD8 transmembrane domain, CD28 and CD3 costimulatory signaling molecules, CD3ζ signaling endodomains. To generate CARs expressing anti-human TIGIT scFv, T2A peptide sequences were intercalated among the second-generation CAR genes. The pMDL-MSLN-CAR-based lentiviral plasmid and two packaging plasmids, pMD-gag-pol and pMD-VSVG, were co-transduced into HEK293 cells in 75 cm^2^ flask at a ratio of 4:3:1, with a total amount of 24 μg. Lentivirus-rich supernatants were collected 48 h, 72 h and filtered through a 0.22 μm filter. Lentiviral vectors supernatants concentrated by ultracentrifugation at 25,000 ×g, 4°C, for 2 h and preserved at −80°C.

### T cell isolation and retroviral transfection

PBMCs from healthy donors and ovarian cancer patients were isolated by Lymphoprep (Stemcell, Canada), and then T cells were isolated from the cells by negative selection using EasySep™ Human T Cell Isolation Kit (Stemcell, Canada). Then activated using anti-human CD3 and CD28 microspheres (Miltenyi, Germany) at a 1:1 bead to cell ratio on day 0. Purified T cells were cultured in 5% FBS X-VIVO Serum-free Hematopoietic Cell Medium (Lonza, Switzerland) supplemented with recombinant human IL-2 (300 IU/mL). Detection of the CAR-T cell positive rate and detection of cell phenotype was performed following lentivirus infection and continuous culture for 48 h after T cell isolation. 1×10^6^ T cells were inoculated into a 24-well plate and 100 μL of lentivirus concentrate were added. T cells were expanded for 2 weeks before downstream experiments. Using CAR-T cells, paired (from same donor) untransduced T cells, activated and cultured for equivalent time, served as control T cells.

### Flow cytometry

All samples were acquired on a CytoFLEX S (Beckman Coulter, Indianapolis, IN), and data was analyzed using Kaluza 2.1 Flow Analysis. Software (Beckman Coulter Life Sciences). Staining for cell surface markers was carried out by incubating with antibodies for 30 min on ice. Antibodies involved in this study included Recombinant PE Anti-Mesothelin antibody(Abcam, Grand Island, NY), mouse IgG1 Isotype Control(R&D, Minneapolis, MN, USA), mouse F(ab)2 IgG (H+L) APC-conjugated Antibody (R&D, Minneapolis, MN, USA), human CD155/PVR PE-conjugated Antibody (R&D, Minneapolis, MN, USA), human TIGIT APC-conjugated Antibody (R&D, Minneapolis, MN, USA), CD226 (DNAM-1) Monoclonal Antibody, APC (eBioscience, San Diego, CA), mouse anti-human CD4-PE (BD, San Diego, CA), mouse anti-human CD8-APC (BD, San Diego, CA), mouse anti-human Foxp3-APC (BD, San Diego, CA). APC anti-mouse CD279 (PD-1) Antibody (biolegend, California, USA), PE anti-mouse CD197 (CCR7) Antibody (biolegend, California, USA), APC anti-human CD45RA Antibody (biolegend, California, USA).

### Western blotting

The cells were lysed in SDS buffer (Invitrogen™, Waltham, MA, USA) containing a protease inhibitor cocktail (PMSF) in accordance with the manufacturer’s protocol. Each sample was sonicated 4 times for 15 second intervals, with at least 15 seconds rest on ice in between successive sonication periods, before being boiled for 5 minutes at 95°C. Cell lysates were separated by SDS-PAGE, transferred to nitrocellulose membranes under the appropriate conditions, and blotted for the following antigens: total human CD155/PVR Antibody (R&D, Minneapolis, MN, USA), GAPDH (Upstate, 05-423) HRP-conjugated mouse-anti-myc tag antibody and HA-Tag (6E2) Mouse mAb (Cell Signaling Technology, Danvers, Massachusetts, USA). Each experiment was repeated at least 3 times. Blots were quantified using ImageJ image analysis software.

### Immunofluorescence

Immunofluorescence was used to analysis the expression of CD155 in tumor cells (Hela and Skov3). The slides with cells were fixed with 4% paraformaldehyde (Boston Bioproducts) for 15 min. Normal goat serum was added to the slides were sealed at room temperature for 30 min. Each slide was dropped with enough Human CD155/PVR Antibody (R&D, Minneapolis, MN, USA) and placed in a wet box for incubation at 4°C overnight. After incubation, sample were washed and incubated with Goat Anti-Mouse IgG H&L (Alexa Fluor^®^ 594) (Abcam, Grand Island, NY) for 60 min at room temperature. The samples were stained DAPI (R&D, Minneapolis, MN, USA) and incubated for 5 min. Then PBST was washed for 5 min, 4 times to remove the excess DAPI. Dry the liquid on the slipper with absorbent paper, seal the slipper with sealing liquid containing anti-fluorescence quench agent, and observe and collect the image under Laser scanning confocal microscope (LSCM).

### Real Time Cytotoxicity Assay (RTCA)

Tumor cells (2×10^4^) were plated in a 96-well. After 24 h, effector T cells were added into the unit at various effector (T cells, anti-MSLN CAR-T cells, anti-MSLN CAR-T cells +anti-α-TIGIT and MT CAR-T cells)/target cell (E/T) ratios (8:1, 4:1, 2:1, and 1:1). Using the impedance-based Real Time Cytotoxicity Assay, RTCA (ACEA, San Diego, CA), the kinetics of tumor cell lysis was evaluated over 80 h. Impedance was measured at 15-min intervals. The impedance-based cell index for each well and time point was normalized with the cell index before adding T or CAR-T cells. The kinetics of cell lysis was evaluated as the change in normalized cell index over time.

### LDH release assay

Tumor cells (2×10^4^) were plated in a 96-well, resistor-bottomed plate in triplicate. After 24 h, effector T cells were added into the unit at various effector (T cells, anti-MSLN CAR-T cells, anti-MSLN CAR-T cells +anti-α-TIGIT and MT CAR-T cells)/target cell (E/T) ratios (8:1, 4:1, 2:1, and 1:1) at 37°C for 4 h. LDH in the culture medium was measured by using commercial kits in 96-well enzyme immunoassay plates according to the manufacturer’s instructions.

### Luciferase report assay

Tumor cells (2×10^4^) were plated in a 96-well. After 24 h, effector T cells were added into the unit at various effector (T cells, anti-MSLN CAR-T cells, anti-MSLN CAR-T cells +anti-α-TIGIT and MT CAR-T cells)/target cell (E/T) ratios (8:1, 4:1, 2:1, and 1:1) at 37°C for 6 h. The above 96-well plate was centrifuged at 1000 ×g for 5 min and some of the supernatant was removed. Then adding 100 μL of luciferase substrate to each well, mix by blowing, and leave for 3 min. The above mixture was transferred to a white 96-well plate and assayed on the machine. Tthe cytotoxicity is relatively to tumor cells grown without T cells (Control group). The relative cytotoxicity rate was calculated as follows: relative cytotoxicity (%) = [Control well OD- (experimental well OD - blank well OD)/(control well OD- blank well OD)]× 100%.

### Enzyme-Linked Immunosorbent Assay (ELISA)

For *in vitro* trials, CAR-T cells (2×10^4^) were co-cultured with tumor cells (2×10^4^) in 96-well plates without the addition of exogenous cytokines. Following 24 h of coculture at 37°C, supernatant was collected and cytokines (IFN-γ, IL-2, TNF-α) were measured by ELISA in accordance with the manufacturer’s instructions (R&D, Minneapolis, MN, USA). For *in vivo* trials, 100 μL of peripheral blood was collected from the treated mice at 4°C overnight and centrifuged for 10 min (1000 ×g) to collect the supernatant. Cytokines in blood serum (IFN-γ, IL-2, TNF-α) were analyzed by ELISA assay, according to the manufacturer’s instruction (R&D, Minneapolis, MN, USA). The content of TIGIT scFvs tested by ELISA analysis. Human TIGIT Protein, His Tag (HPLC verified) (Acro, Delaware, US) were encapsulated in a 96-well enzyme-labeled plate and spent the night at 4°C. The TIGIT antigen was diluted to 100 μg by coating diluent, 100 μL was added into each pore at 4°C for 24 h. 5% calf serum was sealed at 37°C for 40 min. The diluted sample was added into the enzyme-labeled reaction hole at least two holes, 100μL per hole, at 37°C for 60 min. Anti-HA-Tag Mouse mAb was added to connect with HA-Tag in TIGIT scFv. 200 μL TMB color solution per hole was incubated at room temperature for 10 min and then read at 450 nm.

### *In vivo* xenograft models

Female B-NDG mice (4 weeks) were purchased from Biocytogen (Beijing). All procedures were conducted in conformity with guidelines of the National Institutes of Health and Institutional Animal Care and Use Committee. And the animal experiments were approved by the Nanjing Normal University Animal Faculty. Mice were maintained under specific pathogen-free conditions for 3 days, then an equal number of Hela^CD155^ cells (5×10^6^/per mouse) were subcutaneously implanted on the right of the same B-NDG mice, respectively. The progression of xenograft tumors was monitored every three days through the measurement of the length (L) and width (W) of tumors using a digital Vernier caliper, and the tumor volume (V) was calculated as V= (L×W^2^)/2. The mice were randomly divided into 4 groups when the mean of tumor volumes reached 100 mm^3^ mice and different groups were treated with intravenous infused of 5×10^6^ cells (T cells, anti-MSLN CAR-T cells, anti-MSLN CAR-T cells +anti-α-TIGIT and MT CAR-T cells). Mice were given TIGIT antibody (100 μg/per mice) (Biointron, Taizhou, China) on days 0, 7 and 14 post effector cells inoculation, for a total of three doses. Collect blood from mice before execution for used to detect cytokine release levels *in vivo*.

### Copy number of CAR gene in mice

100 μL mice blood were collected every 7 days and DNA was extracted from FastPure Blood DNA Isolation Mini Kit following manufacturer’s instruction (Vazyme, Nanjing, China) CAR DNA was quantified by real-time PCR using primers WPRE-F 5’ GGCACTGACAATTCCGTGGT 3’, WPRE-R 5’ AGGGACGTAGCAGAAGGACG 3’. ChamQ SYBR qPCR Master Mix (High ROX Premixed) (Vazyme, Nanjing, China). The samples were measured in a CFX384 Touch Real-Time PCR Detection System (Bio-Rad). All samples were tested at least in triplicates.

### Immunohistochemistry (IHC)

Tissues were fixed with formalin and embedded in paraffin until further processing. Then 3-mm-thick sections were deparaffinized and treated with a heat-induced antigen IHC Tek epitope retrieval solution (IHC World) for 30 min. Slides were then blocked with tris-NaCl (TNB) blocking buffer (PerkinElmer) and stained with anti-human CD4/8 antibody (Abcam, Grand Island, NY) or anti-human TIGIT antibody (R&D, Minneapolis, MN, USA) in the blocking solution overnight at 4°C. Secondary antibodies were added after rinsing the section for 1 h at room temperature, and the results were visualized with a ChemMate Envision Detection Kit (DakoCytomation). Images were obtained using the 3DHISTECH Panoramic digital slide scanner and the associated CaseViewer software (3DHISTECH).

### Statistical analysis

GraphPad Prism 8.0 software was used to construct all graphs and calculate statistical significance. Kaluza Analysis 2.1 software was used for FCM analysis and to generate plots. For two sets of fold-change measurements, a one sample t-test was used. For comparison of three or more sets of unpaired measurements, one-way ANOVA was performed with Tukey’s *post-hoc* test if all sets were analyzed, or Sidak’s *post-hoc* test if selected relevant pairs were analyzed. Significance from Kaplan-Meier survival curves were calculated with the Log-Rank test. Data is represented as mean ± SD of at least three independent experiments. In all plots, *, *P*< 0.05; **, *P* < 0.01; ***, *P* < 0.001.

## Results

### Construction of anti-MSLN CAR-T and its killing effect on tumor cells

**Figure 1:**
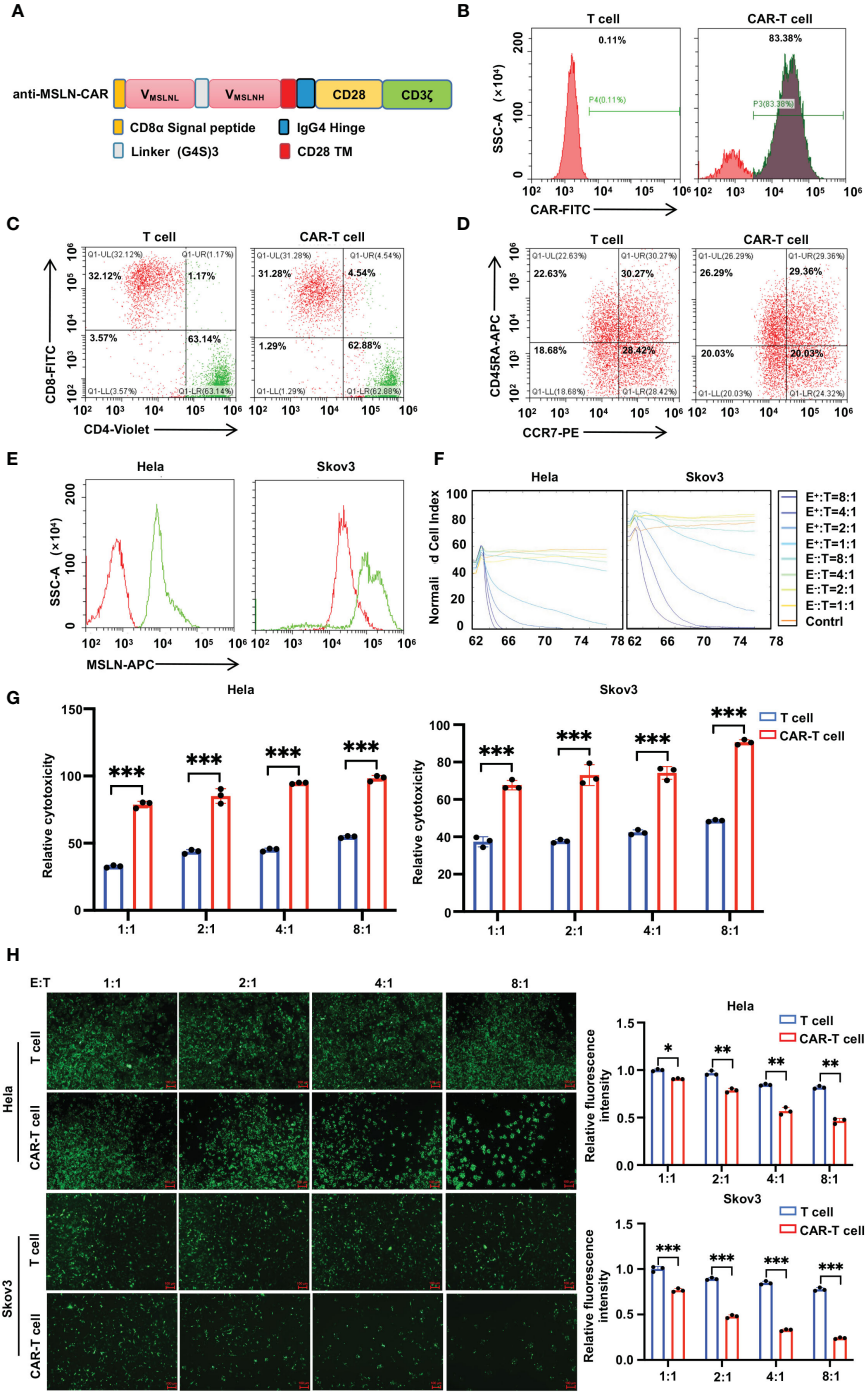
Construction anti-MSLN-CAR-T cells and its killing effect on tumor cells. **(A)** Schemas of MSLN-CARs incorporating different spacers [CD8α Signal peptide, IgG4 Hinge, (G4S)3 Linker, and CD8α™] and costimulatory domains (CD28). **(B)** The transfection efficiency was measured by GFP positive cells using flow cytometric analysis. **(C, D)** Efficient lentiviral transfection of primary human T cells encoding anti-MSLN, with similar CD4/CD8 ratios **(C)**, CD45RA and CCR7 **(D)** in control and CAR transduced T cells. (4 days after lentivirus CAR transfection, the subsets and phenotype of T cells, anti-MSLN CAR-T cells were analyzed by FACS, including the expression of CD4 and CD8.) **(E)** Expression of MSLN in human cell lines were evaluated by FACS. Cells were incubated with anti-MSLN antibody (green) or its corresponding isotype control (red). **(F)** RTCA was used to evaluate the lysis of the indicated tumor cells when treated with T (E^−^) cells or CAR-T (E^+^) cells at a 1:1, 2:1, 4:1, 8:1 effector/target (E/T) ratios over a 78-h period. **(G)** Luciferase report assay results showed lysis of spheres of target cell cultures in the presence of anti-MSLN CAR-T cells or control T cells at the indicated E/T ratios. **(H)** Lysis of spheres of Hela and Skov3 target cell cultures in the presence of T cells (control), or anti-MSLN CAR-T cells at a 1:1, 2:1, 4:1, 8:1 E/T ratios, subjected to immunofluorescence (IF) analysis. Scale bar: 100 μm. Data is represented as mean ± SD of at least three independent experiments. In all plots, *, *P*< 0.05; **, *P* < 0.01; ***, *P* < 0.001.

To validate the tumor killing effect of CAR-T cells, we first designed and generated anti-MLSN CAR-T cells with a highly efficient second-generation human-derived scFv targeting MSLN, which was screened from a human-derived scFv phage display library (**Figure 1A**). The expression of anti-MSLN scFv in lentiviral vector-transfected T cells was detected using flow cytometry 4 days post-infection to verify the CAR transfection efficiency. The results showed that the proportion of stabilized CAR^+^ cells were approximately 83% 4 days post-infection (**Figure 1B**). The proportion of CD3^+^CD8^+^ T cells and CD3^+^CD4^+^ T cells in anti-MSLN CAR-T and T cells were examined using flow cytometry. It was found that the proportion of CD3^+^CD8^+^T cells in both anti-MSLN CAR-T and T cells was around 32%. The proportion of CD3^+^CD4^+^ T cells was around 60%. No significant differences in the distribution of CD4 and CD8 expression were found between CAR-T or T cells (**Figure 1C**). We also examined the phenotypes of CD45RA and CCR7 cells in anti-MSLN CAR-T and T cells. There were no significant differences found in the distribution of CD45RA and CCR7 expression between CAR-T or T cells (**Figure 1D**). In addition, to detect the expression of MSLN in Hela and Skov3 cells, we collected the cells and incubated them with anti-MSLN antibodies by flow cytometry detection. The results demonstrated that MSLN was elevated and specifically expressed in Hela and Skov3 cells (**Figure 1E**). Then we quantified the anti-tumor activity of our anti-MSLN CAR-T cells *in vitro*. The results showed that anti-MSLN CAR-T cells had a significant killing effect on Hela-luciferase-GFP and Skov3-luciferase-GFP cells (**Figures 1F-H**). These results suggested that anti-MSLN CAR-T cells induced considerable cell lysis in target Hela and Skov3 cells.

### TIGIT antibody enhances the anti-tumor effect of MSLN CAR-T cells

**Figure 2:**
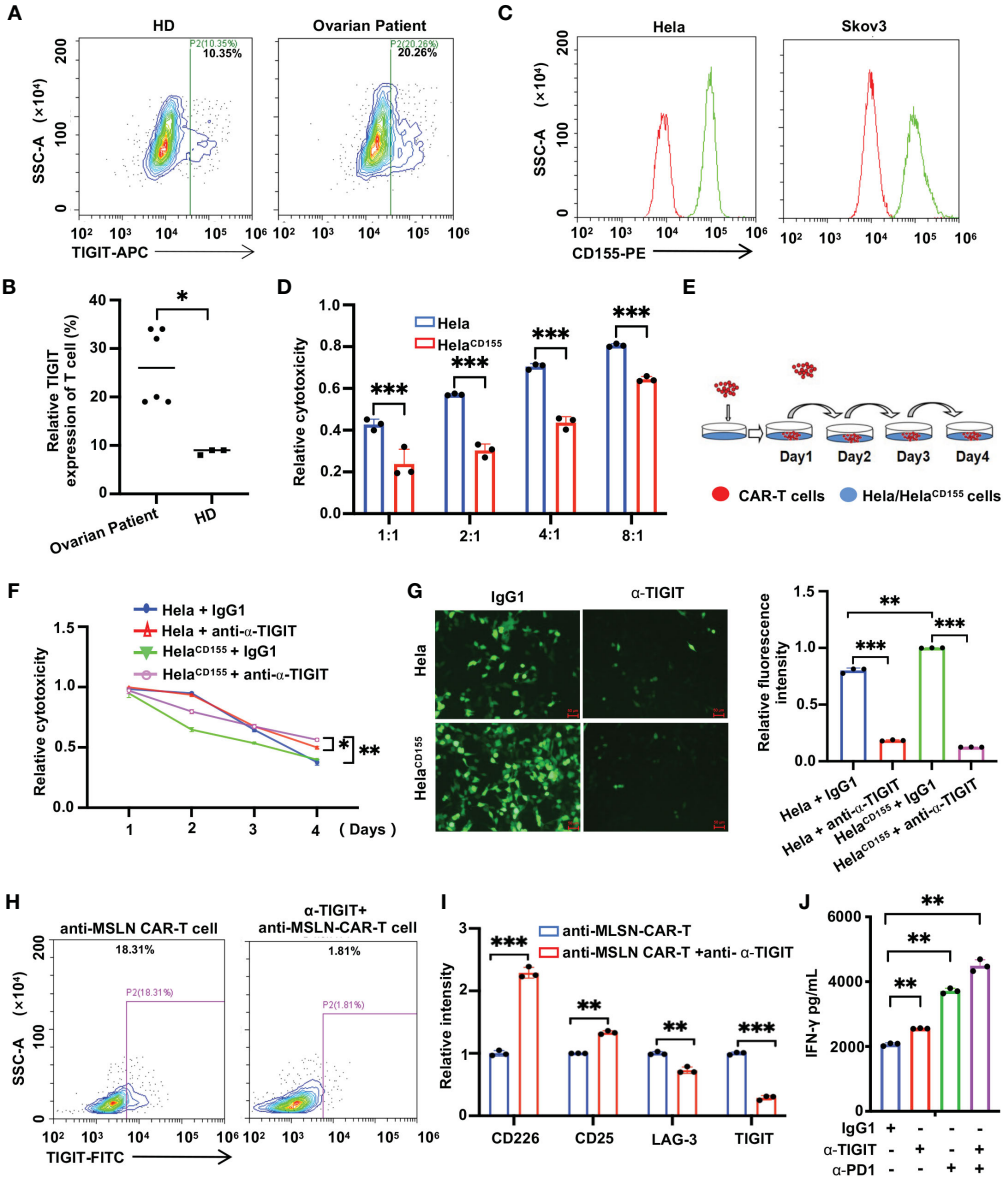
TIGIT antibody enhances the killing effect of anti-MSLN CAR-T cells on tumor cells. **(A)** Flow cytometry determines the expression of TIGIT in activated T lymphocytes from healthy donors or ovarian cancer patients. **(B)** Statistical graph of TIGIT expression in activated T lymphocytes of healthy subjects and ovarian cancer patients. **(C)** Flow cytometry for total CD155 protein levels in human cell lines. **(D)** Luciferase report assay results showed lysis of spheres of Hela/Hela^CD155^ cultures treated anti-MSLN CAR-T cells at the indicated E/T ratios for 6 (h) **(E)** The Schematic diagram of CAR-T continuous killing for 4 consecutive days. **(F, G)** Lysis of spheres of Hela/Hela^CD155^ target cell cultures in the presence of anti-MSLN CAR-T cells, at a 1:1 E/T ratio with or without anti-TIGIT (10^4^ ng/mL) on 4 days, subjected to fluorescein reporting assay **(F)** and IF analysis **(G)**. Scale bar: 50 μm. **(H)** The TIGIT expression was measured in CAR-T cells cocultured with tumor cells in the case of with or without anti-α-TIGIT by GFP positive cells using flow cytometric analysis. **(I)** Anti-MSLN CAR-T cells-treated tumors were harvested 4 h post-treatment at a 1:1 E/T ratio, detecting the phenotype of CAR-T cell activation (CD226 and CD25) and depletion (LAG-3 and TIGIT) by flow cytometry. **(J)** ELISA results showed the IFN-γ secretion levels by anti-MSLN CAR-T combined with IgG1, anti-α-TIGIT, anti-α-PD1, anti-α-TIGIT+anti-α-PD1 for 24 (h) Data is represented as mean ± SD of at least three independent experiments. In all plots, *, *P*< 0.05; **, *P* < 0.01; ***, *P* < 0.001.

TIGIT interacts with CD155, resulting in a reduced anti-tumor effect. Correspondingly, targeting TIGIT can be an effective approach for treating cancers. Thus, we detected TIGIT expression in activated T lymphocytes from healthy subjects and ovarian cancer patients. Flow cytometry results showed that TIGIT^+^ T lymphocytes was about 10% in healthy subjects and 20%-35% in ovarian cancer patients (**Figures 2A, B**). These results indicated that a higher proportion of activated T lymphocytes from ovarian cancer patients expressed TIGIT. Then we found that varying degrees of CD155 expression on the cell surface in Hela and Skov3 cells (**Figure 2C**, **Figure S1A, B**). To detect the influence of TIGIT/CD155 immune checkpoint on MSLN CAR-T cells, we constructed Hela^CD155^, a Hela cell line overexpressing CD155 (**Figure S1C**). The effector cell-induced killing was quantified using a fluorescein reporter assay. The results showed that anti-MSLN CAR-T cells were more effective in killing wild-type Hela cells but less effective in killing Hela^CD155^ cells (**Figure 2D**). Subsequently, we examined the influence of TIGIT on the long-time killing effect of MSLN CAR-T cells. The results showed that anti-α-TIGIT did not influence the killing effect of anti-MLSN CAR-T cells on Hela and Hela^CD155^ cells on Day 1. On day 2, anti-α-TIGIT significantly increased the anti-MSLN CAR-T cell-induced killing on Hela^CD155^ cells (**Figures 2E, F**). On day 4, anti-α-TIGIT enhanced anti-MSLN CAR-T cells continuous killing of both Hela and Hela^CD155^ cells (**Figure 2G**). To further r show the importance of TIGIT on CAR-T cell efficacy, we constructed a Hela cell line knock down CD155, Hela^shCD155^ (**Figure S1D**). Anti-MSLN CAR-T cells had more stronger killing effect on Hela^shCD155^ cells (**Figure S1E, F**). However, the combination of anti-α-TIGIT treatment significantly enhanced the killing effect of CAR-T cells on Hela cells. These findings provide important insights into the role of anti-α-TIGIT in enhancing the sustained killing efficacy of anti-MSLN CAR-T cells against tumor cells over an extended period. Flow cytometry analysis revealed that approximately 18.31% of anti-MSLN CAR-T cells were TIGIT^+^, while the percentage dropped to about 1.81% in the anti-α-TIGIT+anti-MSLN CAR-T cell population (**Figure 2H**). This indicates that anti-α-TIGIT can indirectly or directly block TIGIT on the surface of activated CAR-T cells. Furthermore, we examined the phenotype of anti-MSLN CAR-T cells after co-culture with tumor cells. The results indicated that the anti-MSLN CAR-T cells+anti-α-TIGIT group exhibited a downregulation of immunosuppressive receptors (LAG-3 and TIGIT) compared to the anti-MSLN CAR-T cells group. Conversely, the expressions of T cell activation markers (CD226 and CD25) were upregulated in the anti-MSLN CAR-T cells+anti-α-TIGIT group compared to the anti-MSLN CAR-T cells alone (**Figure 2I**, **Figure S1G**). In addition, we quantified the release of IFN-γ from CAR-T cells. The results showed that both anti-α-PD-1 and anti-α-TIGIT antibodies increased the release of IFN-γ from CAR-T cells. Importantly, when these two antibodies were used together, there was a significant increase in the level of IFN-γ released (**Figure 2J**). In summary, these results indicated that anti-α-TIGIT enhanced the sustained killing effect of anti-MSLN CAR-T cells on tumor cells.

### TIGIT antibody promotes the activation of anti-MLSN CAR/TIGIT NFAT-Jurkat cells

**Figure 3:**
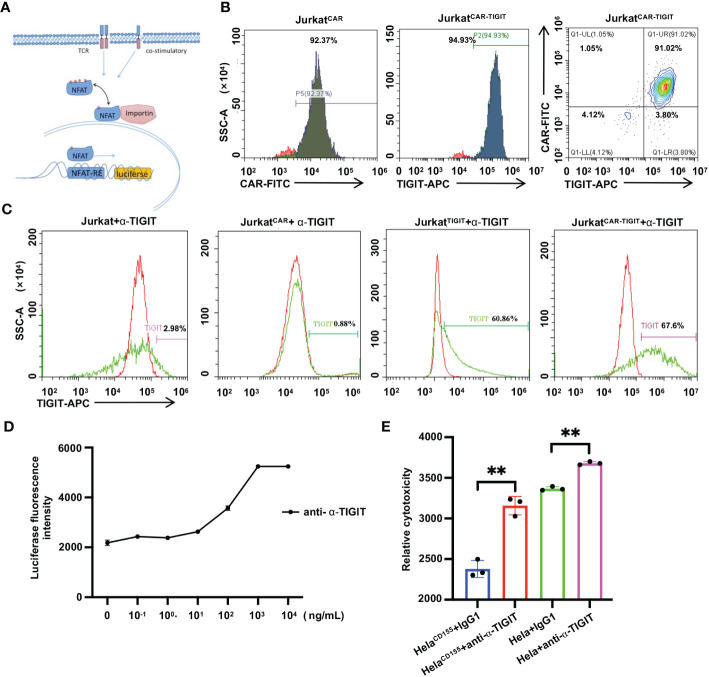
TIGIT antibody promotes T cell activation in NFAT-Jurkat cells report system. **(A)** Schematic diagram of NFAT-luciferin gene structure. **(B)** The transfection efficiency was measured by GFP positive cells using flow cytometric analysis on the 7th day after transfection. **(C)** The binding efficiency of anti-α-TIGIT in NFAT-Jurkat cells was measured by flow cytometry. Cells were incubated with anti-α-TIGIT (green) or its corresponding isotype control (red). **(D)** The degree of activation of anti-MLSN CAR/TIGIT NFAT-Jurkat cells cocultured with Hela^CD155^ for 24 h was shown after treatment with indicated concentrations of anti-α-TIGIT for 24 h **(E)** Luciferase report assay results showed lysis of spheres of target cell cultures treated by anti-MSLN CAR-T cells with or without anti-α-TIGIT (10^4^ ng/mL) at a 1:1 E/T ratio for 24 **(h)** Data is represented as mean ± SD of at least three independent experiments. In all plots, **, *P* < 0.01.

We used Jurkat cells to mimic T cell responses *in vitro* to investigate the effect of TIGIT on T cell phenotypes. T cell activation bioassay is a bioluminescent cell-based assay that overcomes the limitations of existing assays for the discovery and development of cellular therapies designed to induce, enhance, or mimic T cell responses. The assay consists of a genetically engineered Jurkat cell line that expresses a luciferase reporter gene driven by the nuclear factor of activated T cells response element (NFAT-RE) (**Figure 3A**). NFAT-Jurkat cells were transfected with anti-TIGIT, anti-MSLN CAR lentivirus for 24 h and cultured continuously. The results showed that there was 94.93% TIGIT^+^ cells and 92.37% anti-MLSN CAR^+^ cells, which were both high levels (**Figure 3B**). Next, we analyzed the binding of anti-α-TIGIT to NFAT-Jurkat cells. We used a mouse Fc IgG(H+L) APC-conjugated antibody bound to the FC fragment of the anti-TIGIT antibody, thereby indirectly detecting the binding efficiency of the anti-TIGIT antibody to TIGIT on the surface of Jurkat cells by flow cytometry. The results showed that anti-α-TIGIT bound to Jurkat^TIGIT^ at a rate of 60.86% and Jurkat ^CAR+TIGIT^ cells at a rate of 67.6% (**Figure 3C**). In addition, we suggest that Jurkat cell activation status is positively correlated with TIGIT concentration in a certain range (**Figure 3D**). These results showed that the overexpression of CD155 suppressed Jurkat activation level, while anti-α-TIGIT increased Jurkat activation level and reverted the immunosuppression caused by CD155 (**Figure 3E**). In conclusion, anti-α-TIGIT can promote anti-MLSN CAR/TIGIT NFAT-Jurkat cell activation.

### Self-delivery TIGIT-blocking scFv enhances the anti-tumor effects of CAR-T cells *in vitro*

**Figure 4:**
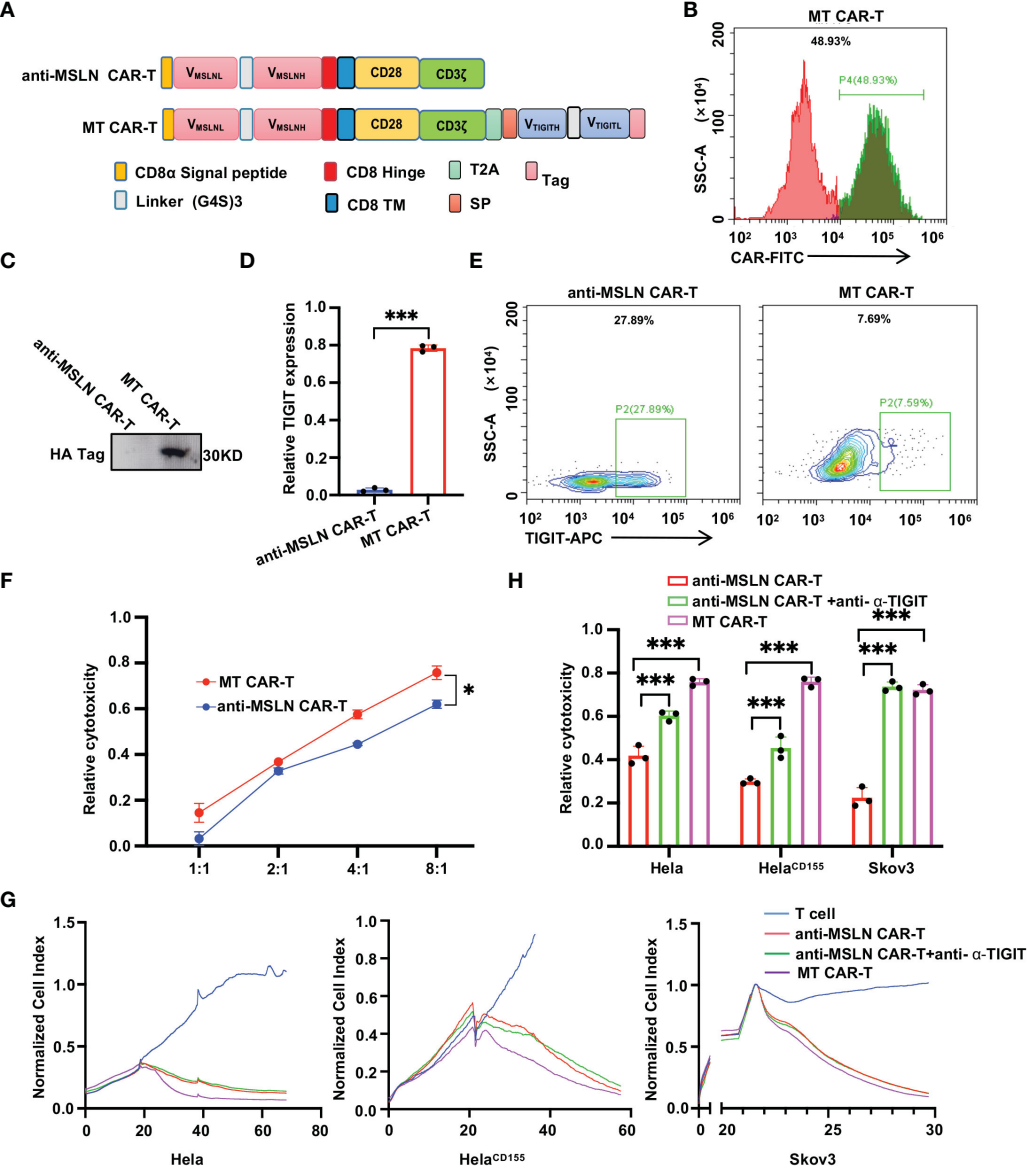
Self-delivery TIGIT-blocking scFv enhances CAR-T cells cytotoxicity to tumor cells. **(A)** Schemas of anti-MSLN-CARs and MT CARs incorporating different spacers [CD8α Signal peptide, IgG4 Hinge, (G4S)3 Linker, CD8α™, T2A peptide sequence, Tag protein and Signal peptide] and costimulatory domains (CD28). **(B)** The transfection efficiency was measured by GFP positive cells using flow cytometric analysis. **(C, D)** Western blot **(C)** and relative quantification **(D)** for TIGIT levels in MT or anti-MSLN CAR-T cells, with three independent assays. **(E)** Detecting TIGIT on the surface of CAR-T cells by flow cytometric analysis. **(F)** Luciferase report assay results showed lysis of spheres of Hela^CD155^ cultures treated by MT cells or anti-MSLN CAR-T cells at a 1:1, 2:1, 4:1, 8:1 E/T ratios for 4 h **(G)** RTCA was used to evaluate the lysis of the indicated tumor cells when treated with control T cells, anti-MSLN CAR-T cells, anti-MSLN CAR-T cells+anti-α-TIGIT and MT CAR-T cells, at a 1:1 E/T ratio over a 60-h period. **(H)** Lysis of spheres of target cell cultures in treated by control T cells, anti-MSLN CAR-T cells, anti-MSLN CAR-T cells+anti-α-TIGIT and MT CAR-T cells, at a 1:1 E/T ratio for 4 **(h)** MT CAR-T cells are more effective against multiple cell lines. Data is represented as mean ± SD of at least three independent experiments. In all plots, *, *P* < 0.05; ***, *P* < 0.001.

**Figure 5:**
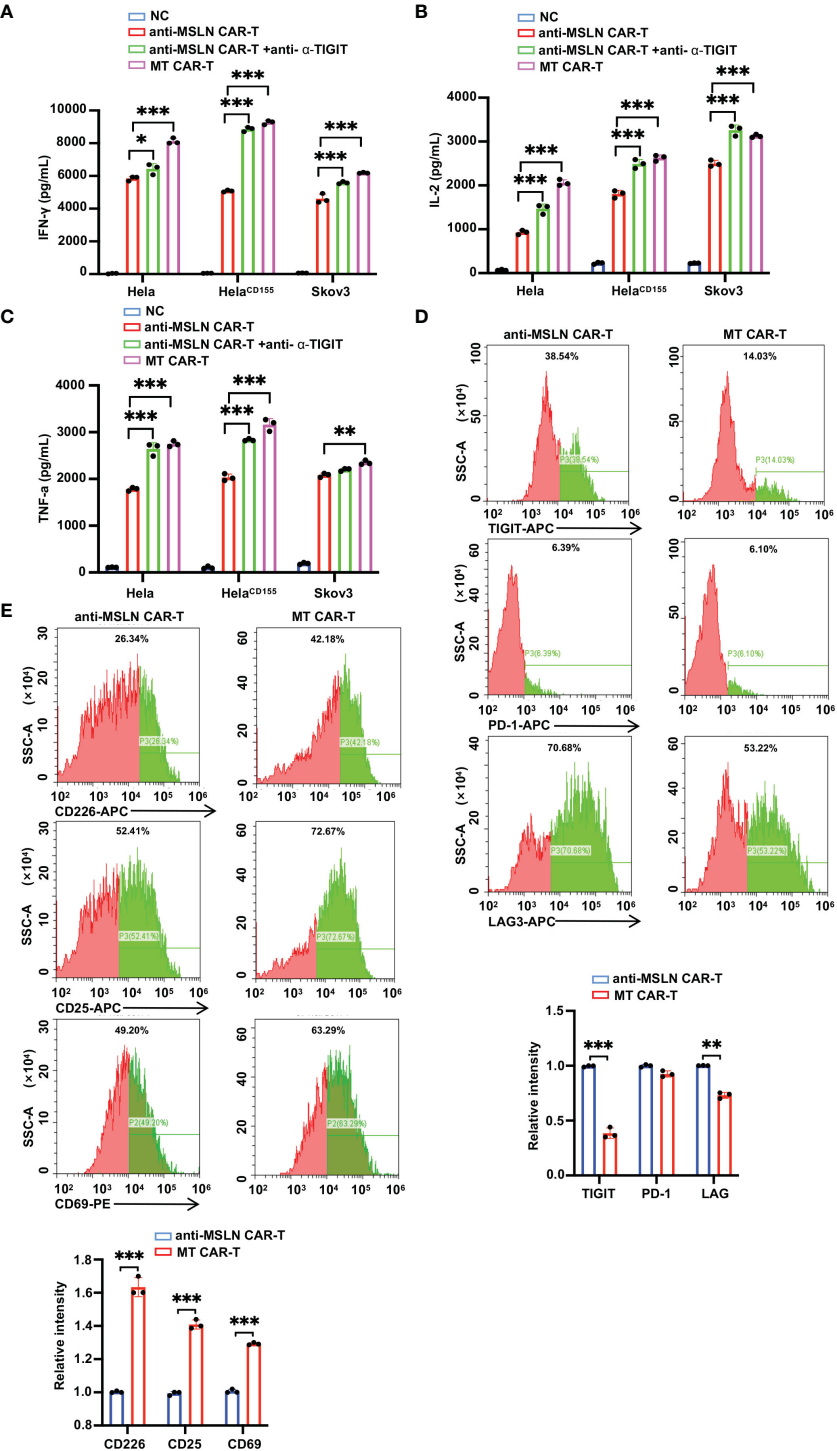
Self-delivery TIGIT-blocking scFv promotes cytokine secretion and modifies the characterization of CAR-T cells. **(A-C)** ELISA results showed the IFN-γ **(A)**, IL-2 **(B)**, and TNF-α **(C)** secretion levels treated by control T cells, anti-MSLN CAR-T cells, anti-MSLN CAR-T cells+anti-α-TIGIT and MT CAR-T cells. **(D)** MT cells or anti-MSLN CAR-T cells-treated tumors were harvested 4 h post-treatment at a 1:1 E/T ratio for 4 h, detecting the phenotype of CAR-T cell depletion (TIGIT, PD-1, and LAG-3) by flow cytometry. **(E)** MT cells or anti-MSLN CAR-T cells-treated tumors were harvested 4 h post-treatment at a 1:1 E/T ratio, detecting the phenotype of CAR-T cell activation (CD226, CD25 and CD69) by flow cytometry. Data is represented as mean ± SD of at least three independent experiments. In all plots, *, *P*< 0.05; **, *P* < 0.01; ***, *P* < 0.001.

Although CAR-T cells have made important advances in the treatment of multiple tumors, there are multiple adverse effects on clinical treatment. Improving the efficacy and safety of CAR-T cell therapy by modifying the structure of CARs is a promising strategy. Thus we introduced the MT CAR gene into T cells by gene transfection to make T cells express secretory TIGIT scFvs, which might block TIGIT on the surface of T cells. Then we designed and generated anti-MLSN CAR-T cells with self-delivery of TIGIT-neutralizing scFv, as shown in **Figure 4A**. The anti-α-TIGIT scFv expression of T cells after lentiviral vector transfection was detected by flow cytometry 4 days after transfection to verify the CARs transfection efficiency. The results showed that there was 48.93% CAR^+^ in MT CAR-T cells after lentiviral transfection (**Figure 4B**). Western blot analysis showed that there was almost no expression of TIGIT scFvs in anti-MSLN CAR-T cells but significant in MT CAR-T cells (**Figure 4C**). In addition, ELISA analysis also indicated that anti-MSLN CAR-T cells had almost no expression of anti-α-TIGIT scFv, while MT CAR-T cells had a high expression of scFvs (**Figure 4D**). We performed a flow cytometry assay to validate TIGIT on the surface of T cells (**Figure 4E**). These results revealed that the expressed anti-α-TIGIT scFv blocked TIIGIT on MT CAR-T cells relative to anti-MSLN CAR-T cells. We quantified the anti-tumor activity of MT CAR-T cells *in vitro*. The results showed that MT CAR-T cells had a great killing effect on Hela, Hela ^CD155^ and Skov3 cells (**Figures 4F, G**). Then, we examined the tumor-killing effect of anti-MSLN CAR-T and MT CAR-T cells by LDH release level. The results showed that the anti-α-TIGIT scFv-expressing group had a better tumor-killing effect compared to the antibody and IgG groups in Hela, Hela^CD155^ and Skov3 cells (**Figure 4H**). Furthermore, the release of IFN-γ, IL-2 and TNF-α was detected using ELISA Kit. Consistently, an increase in cytokine production was observed in the supernatant of MT CAR-T cells and MSLN^+^ tumor cells (**Figures 5A–C**). In addition, we examined the anti-MSLN CAR-T and MT CAR-T cell phenotype after co-culture with tumor cells (**Figures 5D, E**). MT CAR-T cells showed a downregulation of the immunosuppressive receptors (TIGIT, PD-1, and LAG-3) compared to those in the normal anti-MSLN CAR-T cells. In contrast, the expressions of T cell activation markers (CD226, CD25 and CD69) were upregulated in MT CAR-T cells compared with those in anti-MSLN CAR-T cells. These results indicated that self-delivery TIGIT-blockading scFv enhanced the efficacy of anti-tumor function of CAR-T cells on solid tumor cells *in vitro*.

### Blocking TIGIT enhances CAR-T therapy *in vivo*

**Figure 6:**
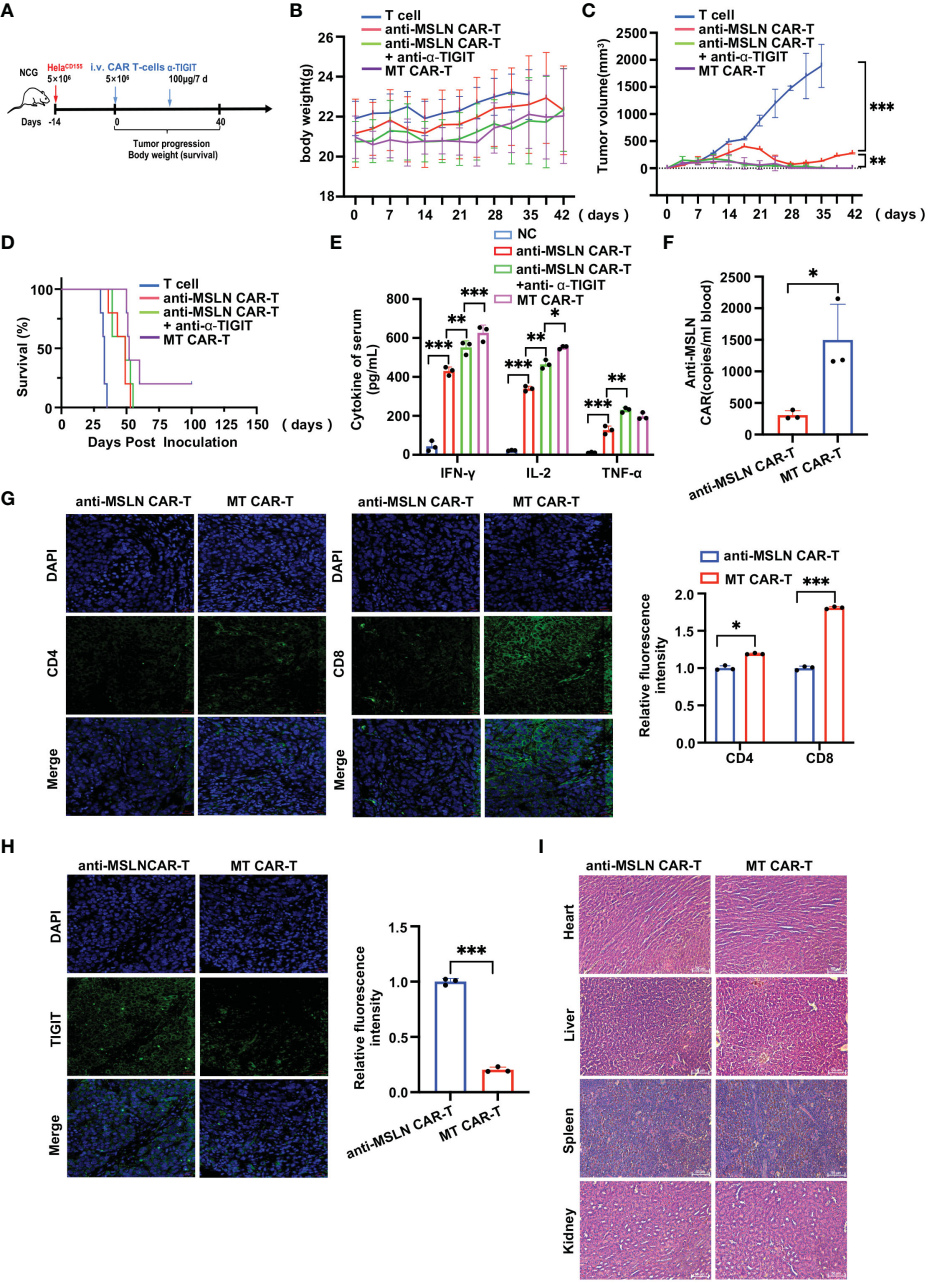
Self-delivery TIGIT-blocking scFv enhances anti-tumor effects of CAR-T cells *in vivo*. **(A)** Treatment scheme used in the Hela^CD155^ xenograft model treated with CAR-T cells and anti-α-TIGIT. B-NDG mice were treated with 5×10^6^ CAR-T cells/mice and anti-α-TIGIT 100 μg/7d. **(B)** Mice body weights monitored during treatment. **(C)** Data showing the tumor volume (mm3) change trend of B-NDG mice in 4 different treat groups. **(D)** Kaplan-Meier survival curve was performed 100 days after Hela^CD155^ cells infused. Mice treated with MT CAR-T cells had a significantly longer survival probability in comparison with mice treated with control T cells, anti-MSLN CAR-T cells, anti-MSLN CAR-T cells+anti-α-TIGIT and MT CAR-T cells. **(E)** ELISA results showed the IFN-γ, IL-2, TNF-α secretion levels in mice blood treated by control T cells, anti-MSLN CAR-T cells, anti-MSLN CAR-T cells+anti-α-TIGIT and MT CAR-T cells. **(F)** Detection of anti-MSLN CAR expression in peripheral blood of mice. **(G)** MT and anti-MSLN CAR-T treated tumors were harvested 3 days post-treatment, subjected to IF analysis for CD4/CD8. Scale bar: 50 μm. **(H)** The TIGIT IF staining were performed in tumors from the resected Hela^CD155^ tumors after MT or anti-MSLN CAR-T cells treated. Representative images of staining intensity are shown. Scale bar, 50 μm. **(I)** MT cells or anti-MSLN CAR-T cells treatment without significant non-target organ damage. Scale: 50 μm. Data is represented as mean ± SD of at least three independent experiments. In all plots, *, *P* < 0.05; **, *P* < 0.01; ***, *P* < 0.001.

Lastly, to determine whether blocking TIGIT could enhance the anti-tumor effect of anti-MASL CAR-T cells *in vivo*, six-week-old B-NDG mice were reared for subcutaneous infused of Hela^CD155^ cells for tumorigenesis *in vivo*. The protocol of specific treatment is shown in **Figure 6A**. The body weight and tumor size of mice were constantly monitored during the treatment (**Figures 6B, C**). There was no significant difference in tumor size between mice in the MT CAR-T cells treatment, anti-MSLN CAR-T cells ± anti-α-TIGIT, and T cells. The tumor growth rate of mice with the CAR-T cells treatment slowed down compared to the one with T cells treatment. The anti-MSLN CAR-T cells showed signs of tumor recurrence in the late stage of the experiment, while the MT CAR-T cells and anti-MSLN CAR-T ± anti-α-TIGIT mice showed no tumor recurrence. MT CAR-T cells treated mice exhibited longer survival times (**Figure 6D**). Peripheral blood samples from mice 3 days after treatment were collected for ELISA to detect serum levels of cytokines (IFN-γ, IL-2 and TNF-α) (**Figure 6E**). The result showed that the mice treated with the CAR-T cells group had higher cytokine level than the T cell treatment group. Meanwhile, higher cytokine levels were detected in the peripheral blood of mice treated with MT CAR-T cells than those treated with anti-MSLN CAR-T ± anti-α-TIGIT. These results indicated that MT CAR-T cells were beneficial to tumor regression. In addition, it was found that there was higher MT CAR expression in the peripheral blood of mice (**Figure 6F**). The results then showed that in the tumor tissues, the group of MT CAR-T cells treatment showed higher CD4 and CD8 infiltration compared to the group of anti-MSLN CAR-T cells (**Figure 6G**). Furthermore, TIGIT expression was lower in tumor tissues with the treatment of MT CAR-T cells (**Figure 6H**). No non-target organ tissue damage was detected in the heart, liver, spleen, and kidney (**Figure 6I**). In summary, these results indicated that self-delivery TIGIT-blocking scFv could enhance the anti-tumor function of CAR-T cells on solid tumors *in vivo*.

## Discussion

**Figure 7:**
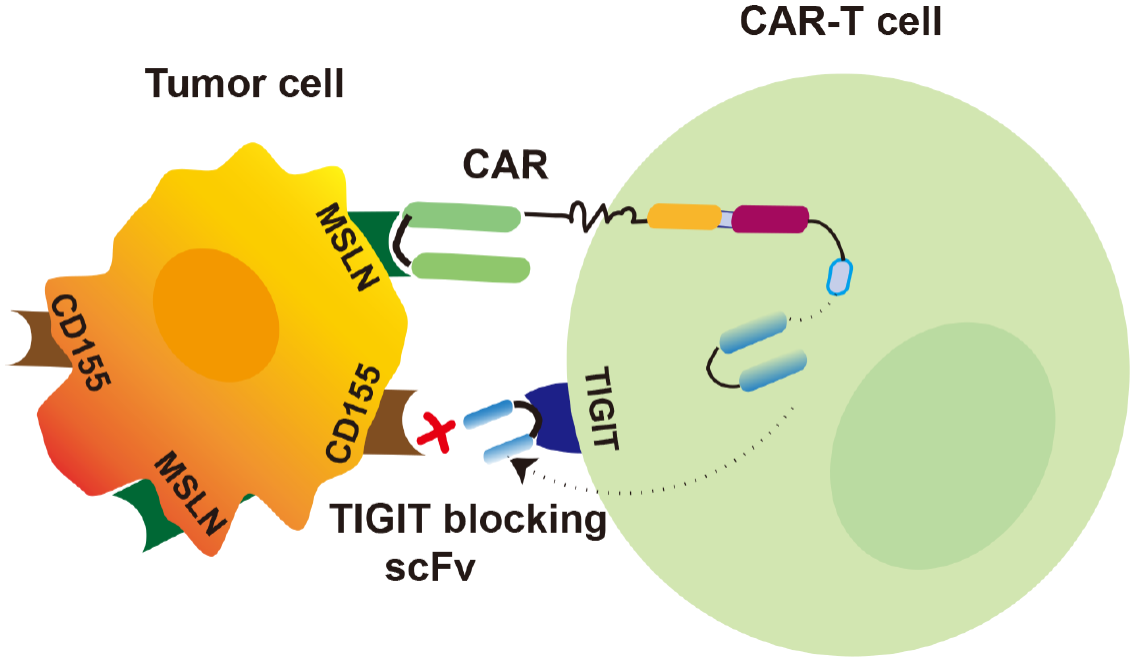
Diagram illustrating the augment of CAR-T cell therapy on tumors by blocking TIGIT. In the proposed model, secreted TIGIT-blocking scFv prevents the interaction of TIGIT on CAR-T cells with CD155 on tumor cells. CAR-T cells specifically recognize the tumor cell surface antigen MLSN to kill tumor cells. Blocking TIGIT enhances anti-tumor immunity of CAR-T cells.

CAR-T cell therapy is in full swing due to their rapid onset of action, high remission rates and long duration of remission compared to traditional biological drugs (29). In contrast, one of the main reasons for non-response or weak response to CAR-T cell therapy is poor T cell expansion and reduced sustained T cell killing capacity (30). Ineffective CAR-T treatment is due to immunosuppression of TME (31). The numerous immune inhibitory sites in the TME pose difficulties for tumor killing, making the combined use of immune checkpoint inhibitors of great interest. TIGIT, as an immune checkpoint, interacts with CD155 expressed on the surface of tumor cells thereby inhibiting the CD8^+^ T cells cytotoxicity in the TME (32). In this study, we demonstrated that blocking TIGIT significantly enhanced CAR-T therapy on solid tumor cells (**Figure 7**).

MSLN is highly expressed in mesothelioma, lung cancer, pancreatic cancer, breast cancer, ovarian cancer, and other cancers (12, 33, 34). Due to its differential expression between cancer and normal tissues and its role in tumorigenesis, MSLN can be considered a potential target. An increasing number of studies have shown that MSLN plays an important role in the promotion of tumorigenesis and progression, although its function in physiological situations is not yet clear (35). MSLN can promote tumor proliferation, metastasis, and resistance to chemotherapy (36). Since MSLN is a highly specific antigen in several cancers, CAR-T therapy has been shown to be a promising strategy for the treatment of these cancers. Here, we constructed anti-MSLN CAR-T cells that significantly induced target cell lysis of Hela and Skov3 cells. However, traditional CAR-T therapy is ineffective in treating solid tumors due to antigen escape, poor tumor infiltration, and immunosuppressive microenvironment (37). Therefore, achieving a broader therapeutic application of CAR-T cells requires a multi-level approach to improve efficacy and safety.

TIGIT acts as an immune checkpoint inhibitory protein that effectively suppresses both innate and adaptive immunity through a variety of mechanisms. It was shown that TIGIT is highly expressed in NK and T cells and associated with CD8^+^ T cell infiltration (20). TIGIT can directly inhibit the functions of CD8^+^ T cell and prevent the clearance of cancer cells (21). CD155 is barely expressed in various normal human tissues but is frequently overexpressed in human malignancies (38). When CD155 on the tumor surface binds to TIGIT on the surface of NK and T cells, it leads to immune escape of tumor cells and the anti-tumor effect is inhibited (39). In addition, TIGIT can further indirectly inhibit anti-tumor immunity by promoting T regulatory cell function of tumor-infiltrating lymphocytes and transmitting inhibitory signals through interaction with CD155 (24). In this study, based on previous studies, we confirmed that blocking TIGIT on the surface of CAR-T cells effectively increased cytokine release in CAR-T cells and enhanced the killing of target tumor cells. Moreover, we have modified the structure of the CAR-T cells to be able to produce TIGIT scFvs (MT CAR-T cells) to achieve the same effect of blocking TIGIT. It was found that MT CAR-T cells could exhibit significant toxicity against solid tumor cells and achieved tumor regression *in vivo*. The modification of CAR-T cells structure reduces the expenditure of treatment and enhances safety of treatment. However, there is a pressing need to continue to evaluate its safety and effectiveness in practical applications in the future.

In summary, this study focuses on the bottleneck of CAR-T cells in immunotherapy of solid tumors. Our study demonstrated that the TIGIT antibody effectively promoted cytokines release and enhanced the killing effects of anti-MSLN CAR-T cells on tumor cells. Moreover, self-delivery TIGIT-blocking scFvs augmented the infiltration and activation of CAR-T cells to enhance tumor regression *in vivo*. CAR-T cells armored for the expression of immunosuppressive protein scFvs may provide a promising strategy for advancing the application of CAR-T and checkpoint blockage therapies in solid tumors.

## Data availability statement

The original contributions presented in the study are included in the article/**Supplementary material**. Further inquiries can be directed to the corresponding authors.

## Ethics statement

The animal study was reviewed and approved by Animal Experiential Ethical Inspection form of Nanjing Normal University.

## Author contributions

ZH, JL, and ZG designed the study. FY, FZ, FJ, and JC contributed to the analysis and interpretation of data. JL, ZC, and ZG contributed to the administrative, technical, or material support. FY, FZ, ZH, and ZG drafted the article. ZH and ZG were responsible for the critical revision. All authors contributed to the article and approved the submitted version.
