# Supplementary material for: Self-delivery of TIGIT-blocking scFv enhances CAR-T immunotherapy in solid tumors
Source: Front Immunol. 2023 Jun 9;14:1175920. doi: 10.3389/fimmu.2023.1175920 (PMC10287952; doi:10.3389/fimmu.2023.1175920)
Supplement: Supplementary file 1 [file DataSheet_1.pdf]

Additional file.

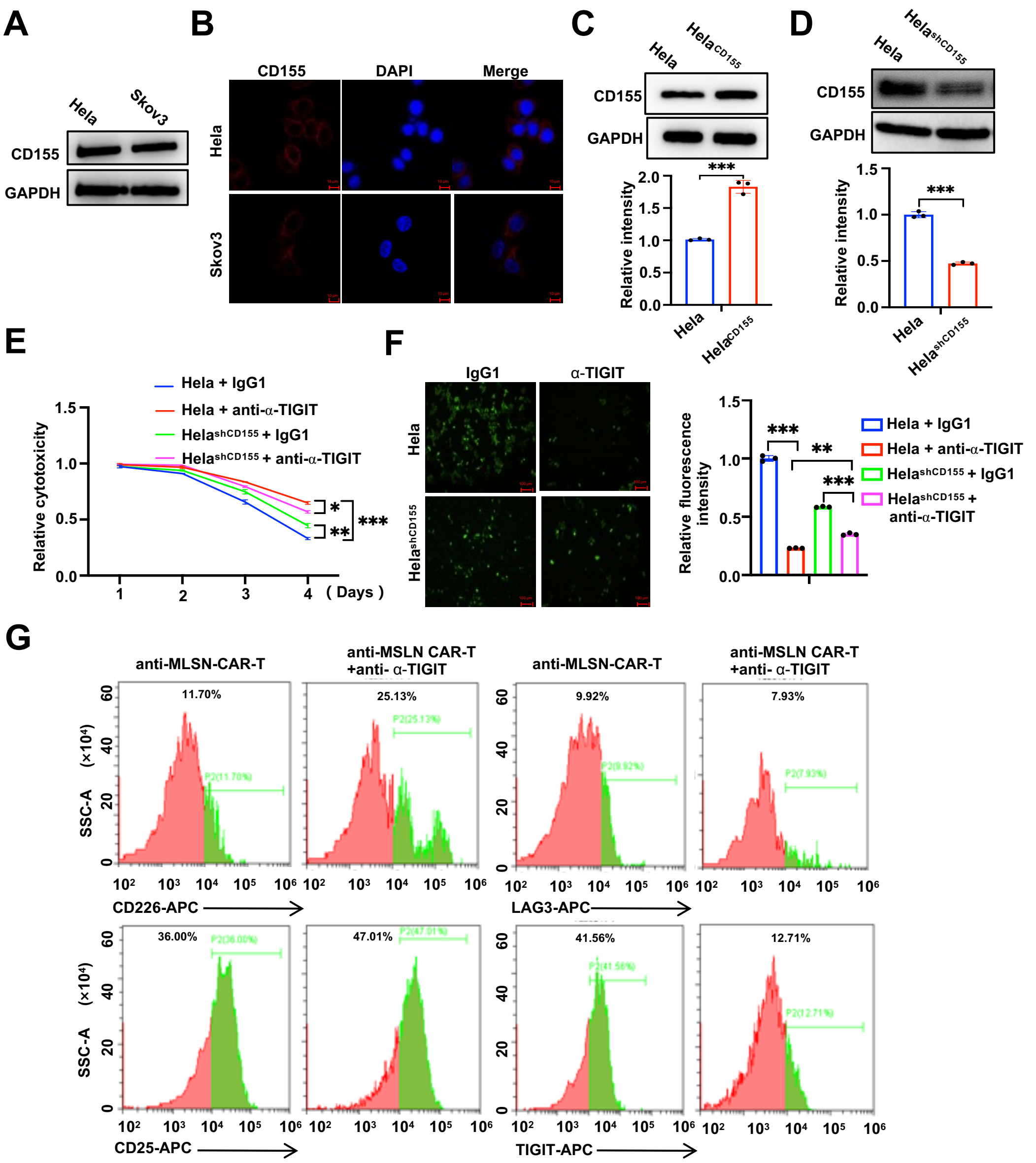

Figure S1 TIGIT antibody enhances the killing effect of anti-MSLN CAR-T cells on tumor cells. **(A, B)** Western blot (**A**) and IF staining (**B**) for total CD155 protein levels in human cell lines. Scale bar: 100 μm. **(C)** Statistical graph of CD155 expression in Hela^{CD155} by Western blot. **(D)** Statistical graph of CD155 expression in $\text{Hela}^{shCD155}$ by Western blot. **(E, F)** Lysis of spheres of Hela /$\text{Hela}^{shCD155}$ target cell cultures in the presence of anti-MSLN CAR-T cells, at a 1:1 E/T ratio with or without anti-TIGIT (10^4 ng/mL) on 4 days, subjected to fluorescein reporting assay **(E)** and IF analysis **(F)**. Scale bar: 100 μm. **(G)** Anti-MSLN CAR-T cells-treated tumors were harvested 4 h post-treatment at a 1:1 E/T ratio, detecting the phenotype of CAR-T cell activation (CD226 and CD25) and depletion (LAG-3 and TIGIT) by flow cytometry. Data is represented as mean ± SD of at least three independent experiments. In all plots, *, $P < 0.05$; **, $P < 0.01$; ***, $P < 0.001$.
